# CONcreTEXT norms: Concreteness ratings for Italian and English words in context

**DOI:** 10.1371/journal.pone.0293031

**Published:** 2023-10-20

**Authors:** Maria Montefinese, Lorenzo Gregori, Andrea Amelio Ravelli, Rossella Varvara, Daniele Paolo Radicioni

**Affiliations:** 1 Department of Developmental and Social Psychology, University of Padova, Padova, Italy; 2 Department of Literature and Philosophy, University of Florence, Florence, Italy; 3 Department of Modern Languages, Literatures, and Cultures, University of Bologna, Bologna, Italy; 4 Department of French, University of Fribourg, Fribourg, Switzerland; 5 Department of Computer Science, University of Turin, Turin, Italy; Hong Kong Baptist University, HONG KONG

## Abstract

Concreteness is a fundamental dimension of word semantic representation that has attracted more and more interest to become one of the most studied variables in the psycholinguistic and cognitive neuroscience literature in the last decade. Concreteness effects have been found at both the brain and the behavioral levels, but they may vary depending on the constraints of the context and task demands. In this study, we collected concreteness norms for English and Italian words presented in different context sentences to allow better control and manipulation of concreteness in future psycholinguistic research. First, we observed high split-half correlations and Cronbach’s alpha coefficients, suggesting that our ratings were highly reliable and can be used in Italian- and English-speaking populations. Second, our data indicate that the concreteness ratings are related to the lexical density and accessibility of the sentence in both English and Italian. We also found that the concreteness of words in isolation was highly correlated with that of words in context. Finally, we analyzed differences between nouns and verbs in concreteness ratings without significant effects. Our new concreteness norms of words in context are a valuable source of information for future research in both the English and Italian language. The complete database is available on the Open Science Framework (doi: 10.17605/OSF.IO/U3PC4).

## Introduction

Word concreteness–the degree to which a word refers to an entity that can be perceived through our senses [1]–has attracted more and more interest to become one of the most studied variables in the literature on psycholinguistic and cognitive neuroscience in the last decade. This semantic representation property indicates whether a word is concrete or abstract and is usually evaluated by participants through Likert scales [1, 2]: concrete words lie herein on one side of the scale and refer to single, bounded, identifiable referents that can be perceived through the senses [3]; abstract words lie on the opposite side of the scale and lack clearly perceivable referents, and rely primarily on interoception (i.e., sensations inside the body [4, 5]). Compared to concrete words, abstract words are acquired later and mostly through language and social interaction (for a recent review, see [6]). Concrete words are also easier to contextualize, while abstract words tend to be more emotionally valenced and less imageable [7]. Furthermore, abstract words are considerably more variable and not organized into a well-defined categorical hierarchy [3, 8, 9]. Participants’ agreement is higher when they produce properties and associations for concrete words compared to abstract words [10]. This could be due to the fact that abstract words generally have a greater number of possible meanings (i.e., polysemy or semantic diversity [11]) compared to concrete words.

This ontological distinction is reflected in their different representations in the human brain. In fact, a growing body of evidence from functional neuroimaging studies supports the idea that the brain areas of the distributed semantic network respond differently to the processing of concrete and abstract words (see, e.g., [12–15]). Therefore, the consensus from these studies is that two main systems underlie the processing of these types of words, with abstract words relying on the verbal language system and concrete words relying on the imagery and perceptual systems [16]. These results could also suggest that semantic representations of abstract and concrete words may be organized according to different dimensions of relation/similarity, which roughly measure the degree to which two words are similar in their meaning [8]. Although abstract words are generally organized by associative relations, concrete words are deemed to be organized by similarity in the sensorimotor experience [9, 17–19]. Surprisingly, the behavior of distributional models (based on the co-occurrence between words in spoken and written language) was largely comparable between concrete and abstract words [20] or accounts better for concrete than abstract words [21, 22].

Differences can also be observed at the behavioral level. Since Paivio et al. [23] published one of the first large-scale databases of word concreteness norms, the so-called *concreteness effect* emerged in a variety of studies of many different cognitive processes. There are hundreds of experimental reports that show that concrete words are processed more quickly and accurately than abstract words. For example, compared to abstract words, concrete words are responded to more quickly in lexical decision tasks [24, 25, but see also 7], are easier to encode and retrieve [26, 27], are easier to make associations with [28], and are more thoroughly described in definition tasks [29].

The relative effects of the concreteness dimension in predicting the performance of participants in different cognitive tasks can also vary depending on the constraints of the context and the demands of the task [4]. For example, in the sentences *’Physics is Alice’s research field’*, *’The magnetic field attracts iron’*, and *’In summer the wheat field is yellow’*, the word *field* has different meanings with three different degrees of concreteness depending on the context in which it appears: in this perspective, each meaning is associated with a given concreteness content. Thus, shades of meaning could be mirrored by subtle differences in the concreteness dimension of words. Indeed, concreteness is not a dichotomous dimension, but rather a continuous dimension of semantic representation, and no clear boundary can be drawn between abstract and concrete words [3, 30].

The *context availability account*, one of the first theories that attempted to explain the concreteness effect, underlined the crucial role of contextual information in the processing of abstract and concrete words [31]. This account posited that the poor performance for abstract words is due to the relative unavailability of associated contextual information in the semantic representation for these words compared with the concrete ones. Indeed, when abstract words are presented with sufficient contextual information, the differences between concrete and abstract words in participant performance during cognitive tasks are reduced [31, 32].

Characterizing the concreteness degree of target words in contexts (operationalized as sentences) is thus particularly important to understand cognitive effects of concreteness; it is also challenging, as it requires integrating both word-specific and contextual information. Recent attempts to predict the concreteness score associated with target words presented in different contexts come from the Natural Language Processing (NLP) community. Specifically, a shared task for systems able to predict the concreteness score of participants for words in context in different languages had been organized in 2020 [33]. The best-performing computational model for estimating the concreteness of a target word presented in context used information derived from behavioral norms (e.g., age of acquisition, affective, and sensorimotor dimensions) as well as context-dependent distributional models, underlining the relevance of information related to the word taken in isolation and to the context in which the word occurs [34]. For this reason, it is compelling to build concreteness norms for words presented in several context sentences.

Databases providing concreteness ratings for words in isolation have been developed in several languages such as, for example, English [1], Italian [2], Croatian [35], Dutch [36] and French [37]. Furthermore, concreteness norms have been developed at the sentence level using metaphors and figurative language (e.g., idioms) more generally [38–43]. A metaphor is, in most cases, a mechanism to quickly deliver some information when an abstract concept (*explanandum*) is explained by referring to something else, which is more directly understood (*explanans*) [44], such as in ‘*Love is a rose*, *but you better not pick it’*. Typically, this second element comes from a more direct physical experience of the real world [42].

Katz et al. [45] were the pioneers in the development of norms for many variables of literal metaphors in English. However, this data set did not collect the concreteness values of the metaphors and did not fully include the original material, preventing its extension (and comparison) to other languages. More recently, other standardized metaphor datasets have been developed that include concreteness ratings of participants in English [46, 47] and Italian [38]. Cardillo et al. [46] collected the concreteness values for each of the content words forming the metaphors and then derived a total concreteness value for the metaphor sentence by averaging their concreteness values. On the contrary, Bambini et al. [38] asked the participants to evaluate the concreteness of the entire metaphorical phrase. In particular, the latter study found a correlation between concreteness and familiarity and difficulty, suggesting that more concrete metaphors were felt more familiar and less difficult to understand.

Following this line of reasoning, Muraki et al. [48] recently collected concreteness ratings for 62,000 English multiword expressions that are recognized as central for both language use and acquisition. Although in this work the same word did not occur in different settings (which is, in contrast, a major trait in the present work), the authors here seem to share our own emphasis on contextual features, as they explicitly mention that multiword expressions also provide contextual information by allowing one to disambiguate between expressions such as, for example, ‘bank account’ and ‘river bank’.

Although all the norms presented above represent a great tool for controlling psycholinguistic variables at the sentence level, they overlook the type of information conveyed by a specific target word presented in different contexts. Due to the crucial effect of contextual information on processing differences between abstract and concrete words, we focused our investigation on the dimension of concreteness.

One main assumption underlying the whole work is that each term may have more associated meanings, and meaning selection is determined based on the context, which, in turn, corresponds to some sort of word meaning disambiguation. That is, the concreteness rating involves a hidden task in which meanings are identified [49], and concreteness is supposed to be a property of word meanings rather than of word forms/terms. While we fully acknowledge the importance of addressing word ambiguity, encompassing both homonymy (where a word form’s meanings have distinct historical origins and are not related) and polysemy (where a word form’s meanings stem from the same lexical source and are related), practical considerations challenge the rigid application of the etymological criterion. Notably, two challenges arise in distinguishing cases of polysemy from homonymy: firstly, words may have uncertain historical derivations, making definitive classification difficult. Secondly, determining the extent to which we should trace the historical evolution of word meanings lacks clear guidance. Moreover, the concept of “relatedness of meaning” does not allow for a strict binary categorization but instead spans over a nuanced and continuous spectrum. In a multitude of cases, native speakers do not reach a consensus on whether certain word meanings are related. Consequently, a clear-cut dichotomy between homonymy and polysemy proves elusive, as the spectrum extends from “pure” homonymy to “pure” polysemy [50]. Given the intricate nature of lexical ambiguity in theory and the practical objectives of our study, which aimed to furnish concreteness values for words in standalone context sentences, we did not consider word ambiguity in the selection of target words. Indeed, we selected our target words from concreteness norms for isolated words in the Italian [1] and English [2] languages in order to compare concreteness ratings for words presented with and without context. Because of this choice and of the general distribution of homonymy and polysemy in the lexicon, our final dataset tilts toward an underrepresentation of homonymous terms relative to polysemous ones.

To our knowledge, we presented the first set of concreteness values for target words in context sentences as material for the CONcreTEXT task, in the framework of the evaluation campaign of NLP and speech tools for the Italian language (Evalita shared task and data for that task can be retrieved from the URLs https://lablita.github.io/CONcreTEXT/ and https://osf.io/j4dz3/, respectively) [33].The present work, introducing the data collection named ’CONcreTEXT norms’ (simply CONcreTEXT hereafter), substantially extends that set of annotated data by increasing the number of annotations for each word. For this reason, these norms were acquired from native speakers of Italian and English (as the universal language used in the research community) in very similar settings across the two languages.

Although the concept measures are in general stable across languages and cultures, language-specific effects can be also present, especially in our case, where words were presented within context sentences. A comparison (even if not direct) between English and Italian data may help to investigate an important issue that has been somewhat overlooked in the relevant literature, namely to what extent the concreteness ratings reflect a universal (or at least, culturally dependent) property of concepts that are stable across languages and to what extent they are instead language specific. Unfortunately, given the lack of sentences overlap between English and Italian, we could compare the data across the two languages only indirectly.

The data set also contains additional lexical and syntactic measures, such as lexical density and syntactic tree depth, which characterize sentences in terms of readability and complexity in both semantic and syntactic accounts. The main strength of this dataset is that the stimuli have been derived from the WikiHow website, and thus they represent natural and ecological examples of real-world usage of the language. Additionally, WikiHow offers articles in a multilingual environment, making this stimuli collection methodology and the relative scores easily extensible to other languages. The target words were chosen from the Italian [2] and English [1] concreteness norms of words in isolation to investigate the context effect by comparing those values with those collected in the current norms. We also explore the relation between concreteness and other linguistic and semantic variables that could influence the processing of words in different contexts.

## Methods

### Participants

The complete sample included 319 native Italian speakers and 362 native English speakers for the Italian and English norms, respectively (see Table 1 for the demographic characteristics of the sample). Italian participants have been identified through the personal networks of researchers, while English participants were recruited through the Prolific platform subject pool (using the Prolific recruitment policy, https://www.prolific.co/). Data collection occurred between May 2020 and November 2020. Some participants took part as volunteers without further compensation (those recruited through the personal networks), while others received a small monetary reward (£1.05 per list of sentences, which is equivalent to £6.50 per hour). All participants were over 18 years old.

**Table 1:** Demographic characteristics of the Italian (IT) and English (EN) samples.

Participants’ information		IT	EN
Total number		319	362
**Gender**	Female	190	243
	Male	112	116
	Other	17	3
**Age**	min	18	18
	max	78	77
	mean	39.5	37.8
	median	35	34
	*SD*	13.54	14.60
**Education**	Primary School	0	1
	Secondary School	7	14
	High School	51	89
	Bachelor Degree	36	155
	Master Degree	109	62
	PhD / Specialist Training	116	41

The study has received approval from the Research Ethics Board (*Comitato di Bioetica dell’Ateneo*) of the University of Turin (Protocol number 179481) and was carried out in accordance with the ethical standards of the Declaration of Helsinki for Human Studies (World Medical Association). The authors did not have access to information that would allow identification of individual participants during or after data collection.

### Materials

To offer ratings comparable with the concreteness norms for word in isolation provided by [2] for Italian (henceforth MONT) and [1] for English (henceforth BRYS), we started our selection of lemmas from these datasets. We took the Italian word list as the starting point since it contains fewer items than the English one (1,121 vs 39,954) and provides the English translation for each term. In this list, verbs were less numerous than nouns (5% vs 69% of the total items); for this reason, we decided to keep all verbs therein. The nouns were randomly chosen among different ranges of concreteness values provided in the MONT norms. Given the cost of the annotation procedure, we selected 150 lemmas from MONT, with the aim of annotating 2 to 6 occurrences for each lemma, for a total of 562 sentences for the Italian and 544 for the English language. The final distribution for verbs and nouns was 38.6% verbs and 61.4% nouns for the English language and 33.5% verbs and 66.5% nouns for the Italian language.

Given our aim to investigate concreteness ratings in context, we extracted the occurrences of the words to be annotated from natural examples. Sentences were derived from the English-Italian parallel section of The Human Instruction Dataset [51], a corpus that collects and organizes articles in machine-readable format from the WikiHow website in 16 languages. The whole Human Instruction Dataset is freely available on Kaggle at https://www.kaggle.com/paolop/ human-instructions-multilingual-wikihow. The corpus provides instructions on disparate topics, describing both concrete actions (for example, boiling tea) or abstract events (for example, how to increase self-esteem). We extracted the articles that were present in both the English and Italian sections, to guarantee a parallel between our English and Italian datasets.

We then manually extracted from the corpus the sentences containing the candidate target words; in doing so, we tried to preserve as many different meanings as possible to ensure a comprehensive coverage of the semantic variation for each target word. Specifically, we uploaded the corpus to an instance of Nosketchengine (http://corpora.dipartimentidieccellenza-dilef.unifi.it/noske/) [52] and selected the candidate sentences from the list of the most salient collocates. The log-Dice metric was used to sort the collocations. Collocates let us grasp the meaning of a specific token even before inspecting the whole sentence, thus facilitating, and speeding up our task. Lemmas that did not occur in the corpus were excluded. After a first selection of 872 sentences for English (518 for nouns and 354 for verbs) and 1,033 for Italian (646 nouns; 387 verbs), the five authors annotated the concreteness of the targets in these sentences with two goals: i) measuring their own inter-annotator agreement, to evaluate the feasibility of the task; and ii) applying a second filtering to the sentences, to account for the variability terms of contexts and target concreteness. We then refined the final set of sentences trying to select meanings (occurring in contexts) as varied as possible and removing ambiguous sentences. e filtered out from the collection those cases in which the target word occurred in idioms and multiword expressions, where word meanings were supposed to differ from the meanings of that word (for example, ‘kick the bucket’). Moreover, when necessary, we modified the sentences to resolve anaphoric references (looking at the entire paragraph of the corpus). Furthermore, in the effort to control the length of sentences, sentences with more than 15 tokens or more than 8 content words were removed or cut. The detailed figures that provide the descriptive statistics of the resulting data are presented in Tables 2 and 3.

**Table 2:** Descriptive statistics of the collected data, illustrating the number of lemmas and sentences in both Italian (IT) and English (EN) subsets.

Target	IT		EN	
	Lemmas	Sentences	Lemmas	Sentences
**Nouns**	94	370	74	334
**Verbs**	52	192	44	210
**Total**	146	562	118	544

**Table 3:** Token counts for both the Italian (IT) and English (EN) subsets.

Total tokens		
	IT	EN
**Total number of tokens**	8,101	7,825
**Sentence length in tokens**		
**Min**	5	5
**Max**	24	26
**Mean**	14.41	14.38
**Median**	15	14
***SD***	3.53	3.97

Finally, the selected sentences (see Table 2) were partitioned into 10 different lists, with a maximum of 62 and a minimum of 58 sentences for list. Each participant could provide concreteness ratings for more than one list of sentences. Each list was used to collect concreteness ratings, including at least two sentences of the same lemma in the same list. When possible, sentences with different grades of concreteness (given our preliminary annotation) were chosen. Each sentence received on average 62.9 (*SD* = 12.15) ratings from 362 raters for the English data and 38.6 (*SD* = 6.55) ratings from 386 raters for the Italian data. Tables 2 and 3 summarize the size of the two data sets.

### Procedure

**Fig 1:**
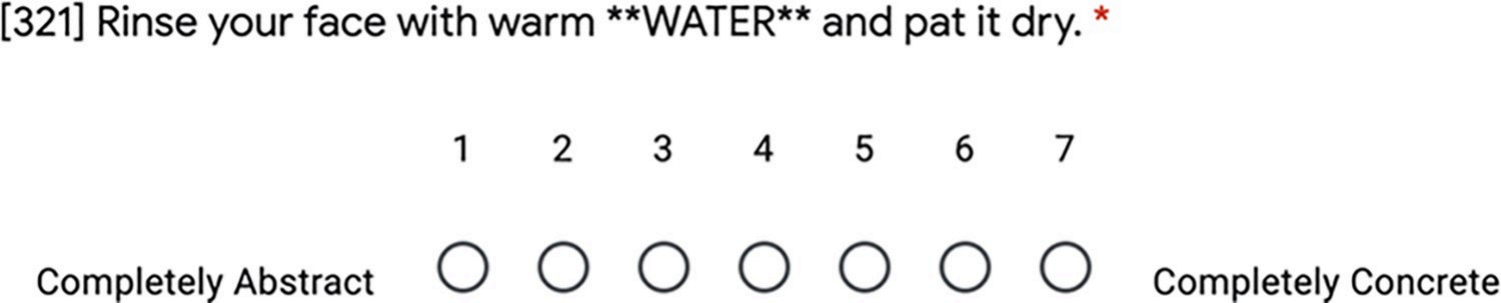
Ratings interface. An example of the rating interface employed in this study has been shown.

An online survey procedure (using Google forms) was devised to ask participants to assign a concreteness score to the target words marked with asterisks (e.g., marked as **WORD**) within a sentence. The scale used ranged from 1 to 7, where 1 corresponds to ’completely abstract’ and 7 to ’completely concrete’. Fig 1 shows an example of a rating item. The instructions emphasized the importance of using the entire range of ratings and explicitly stated that there were no correct answers.

At the beginning of the form, participants were informed about the purpose of the study, the institutions, and the researchers involved, and were asked to give their informed consent. Their demographic information (gender, age, education) was collected. The first four items of the online survey were intended to familiarize participants with the task. We calculated a mean score for each item by averaging the concreteness scores of all participants. The ratings provided by each participant were then compared to the mean rating using the Euclidean distance.

Finally, the ratings provided by a given participant were discarded if their Euclidean distance was greater than two standard deviations from the average of the Euclidean distances featuring all raters. Ratings were collected through 10 lists of sentences, each containing about 60 target terms and as many sentences. Overall, 429 lists were collected for the English language and 371 for the Italian language: 60 and 48 were filtered out (14.0% and 12.9% of all collected answers), based on the above procedure.

## Results

### Database

The normative data in CONcreTEXT include concreteness values for 118 English and 146 Italian words presented in different contexts for a total of 544 English and 562 Italian sentences. Values are derived from judgments provided by 362 English and 386 Italian speakers (see section Participants), and each word in the context was rated by at least 31 participants. There were no missing values, that is all participants provided a rating for all target words under evaluation.

The database includes the full list of English and Italian target words along with their part of speech tag, the index of the word in the sentence, the sentences, aggregated means and standard deviations of the concreteness ratings, the WordNet synset of the target word (if available) and a series of linguistic (lexical and syntactic) variables characterizing the target word and the full context of the sentence. Lexical and syntactic measures have been obtained making use of the Profiling-UD tool [53]. This tool is a complete linguistic analysis pipeline used to compute a linguistic profile of a text or collection of texts, by extracting a wide set of linguistic features. It is based on linguistic annotation and syntactic parsing output from UD-pipe [54]. These linguistic features are considered to be related to readability and text complexity [55], and may have influenced the raters’ judgments.

*WN_synset* is the ID of the word meaning as it is encoded in the meaning inventory of WordNet. WordNet is a lexical database for the English language. Its constructive rationale relies on grouping terms into sets of synonyms (called *synsets*) that are equipped with short definitions and usage examples. WordNet is a broad-coverage semantic network whose relations include several semantic relations between synset elements, such as hyponymy/hypernymy, meronymy/holonymy, antonymy, and others [56]. To retrieve the synsets for the Italian language, we employed the multilingual version of WordNet, Open Multilingual Wordnet [57, 58]. The annotation process is described in the section ‘Comparison with WordNet synsets’.

The *target lexical accessibility* and the *sentence lexical accessibility* are two measures related to the frequency of tokens (word forms). We include these measures based on the assumption that more frequent words are more accessible to the speaker and thus potentially more familiar. We used the Wacky corpora for English and Italian (ukWaC and itWaC [59]) as reference vocabularies, to offer a comparable distribution measure between the two languages. Both corpora count more than 1 billion words and have been collected by scraping the Internet. Given the dimension and heterogeneity of the language they capture, they can be safely considered as reference corpora. Instead of looking at the raw frequency of the lemmas in the vocabularies, we computed average frequency classes and reshaped their distribution into bins by applying a formula like the one proposed by [60]. We scaled the resulting classes in a [0–1] range by dividing the obtained class number by the class number of the most frequent token to offer a finite measure and to facilitate the comparison between the stimuli. The formula we employed is the following:

$${\mathrm{Target}}_{lexACC=}log2\frac{freq(TW)}{freq(LFW)}/log2\frac{freq(MFW)}{freq(LFW)},$$

where MFW is the most frequent word form in the vocabulary, LFW the less frequent and TW is the target word.

*Sentence lexical accessibility* is the average of the lexical accessibility of all the word forms composing the sentence.

*Lexical density* denotes the ratio between content and function words inside a text. We consider as content words those belonging to open classes of parts of speech that carry semantic information: nouns and verbs (and their modifiers), adjectives, and adverbs. This feature is computed as the sum of content words divided by the total number of words in a sentence. Lexical density is typically considered as an indicator of cognitive load [61].

*Sentence length*, *clauses*, and *average tokens per clause* indicate respectively the total number of word forms, the number of clauses composing the sentence, and the average word distribution over the total number of clauses. These are basic measures derived from raw counts but are typically considered as a proxy for lexical and syntactic complexity in traditional readability assessment metrics [62].

The *syntactic tree depth* indicates the longest path from the root of the dependency tree to the deepest leaf. Dependency parsing of sentences results in tree representations in which words are nodes (leaves) and dependency/modification relations are edge labels. This measure is related to the sentence length and is considered as a feature that impacts processing difficulty [63].

The concreteness norms are freely available to the scientific community for noncommercial use in the Open Science Framework repository (https://osf.io/j4dz3/). Table 4 presents descriptive statistics for all variables included in the database.

**Table 4:** Statistics for each variable describing the collected data.

Variable	Italian (mean/*SD*/min/max)	English (mean/*SD*/min/max)
**Concreteness (mean)**	4.269 / 1.522 / 1.44 / 6.96	4.448 / 1.397 / 1.31 / 7
**Concreteness (*SD*)**	1.534 / 0.355 / 0.19 / 2.35	1.613 / 0.393 / 0 / 2.43
**Target lexical accessibility**	0.413 / 0.138 / 0.192 / 0.923	0.411 / 0.115 / 0.192 / 0.692
**Sentence lexical accessibility**	0.257 / 0.036 / 0.163 / 0.388	0.228 / 0.036 / 0.139 / 0.454
**Lexical density**	0.514 / 0.085 / 0.3 / 0.833	0.558 / 0.11 / 0.273 / 0.9
**Sentence length**	15.171 / 3.82 / 5 / 26	14.43 / 3.994 / 5 / 26
**Clauses**	2.295 / 1.04 / 1 / 7	2.351 / 1.06 / 1 / 6
**Average tokens per clause**	7.932 / 4.046 / 2.429 / 26	7.178 / 3.327 / 2.25 / 22
**Syntactic tree depth**	4.016 / 1.017 / 2 / 8	3.697 / 0.978 / 2 / 8

### Descriptive statistics

**Fig 2:**
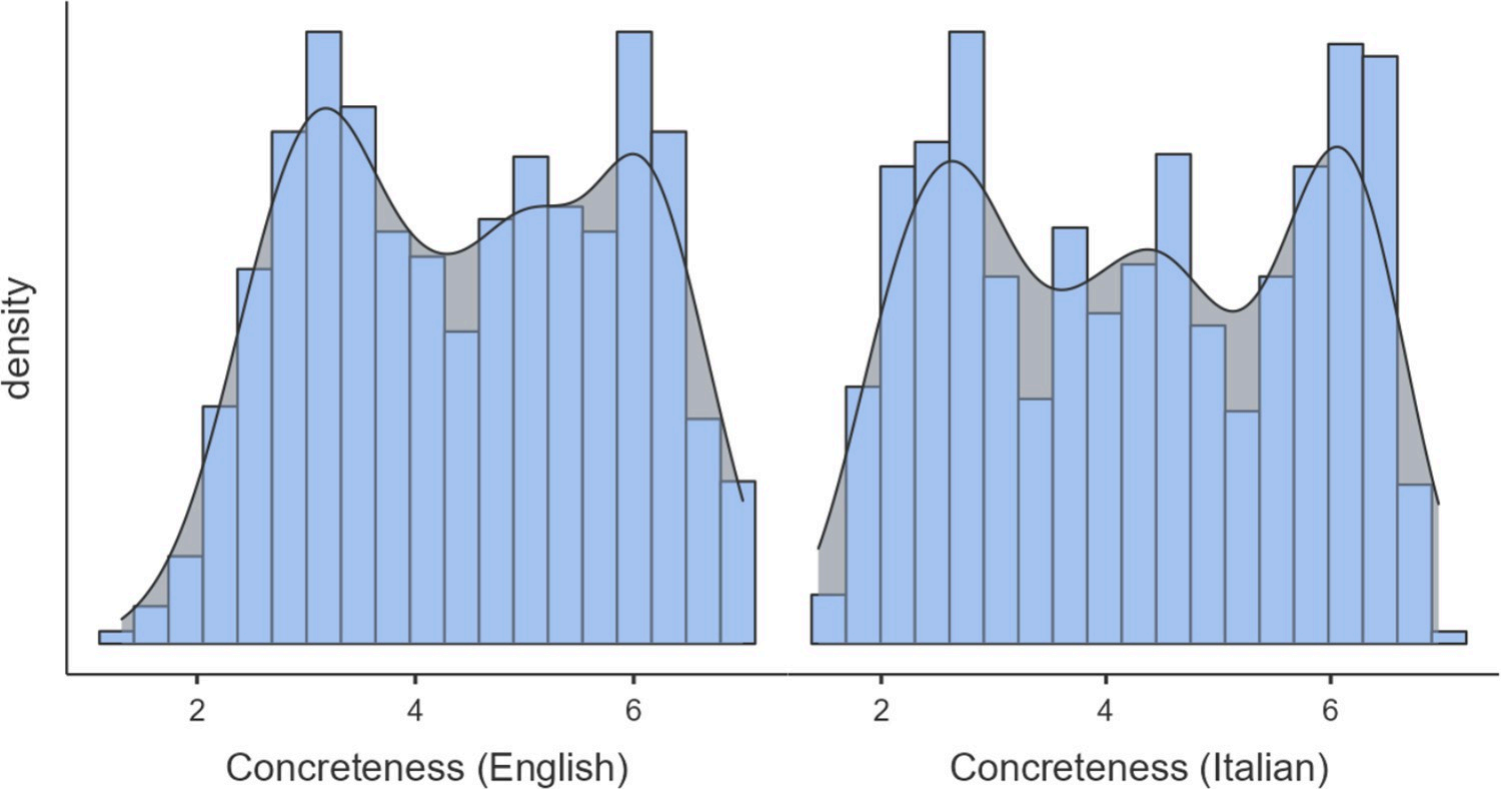
Distribution of the concreteness ratings. Distribution of the mean concreteness ratings for both English (left column) and Italian (right column). The figure shows the estimate of the probability density of the mean concreteness ratings (black line) based on a kernel function.

Fig 2 shows the distributions of the mean concreteness ratings for the English and Italian participants. The two distributions deviated significantly from a normal distribution (Shapiro Wilk test: English: *W* = .912, *p* < .001; Italian: *W* = .941, *p* < .001) with a mean of 3.97 (*SD* = .06, *IQR* = 2) and 4.28 (*SD* = 1.51, *IQR* = 2.88), and data points ranging between 1 and 7, and 1.44 and 6.96, for English and Italian ratings, respectively. Kurtosis was -1.16 for English and -1.33 for Italian. Skewness was .0220 for English and—.0007 for Italian. A bias towards the high range of the scale was observed for both English and Italian, since 58% of the English words and 56% of the Italian words were rated as above the median value (i.e., 4), both percentages being statistically different from the chance level (*p*s < .001, binomial test against 50%). This bias was similar between the English and Italian participants (*χ*2 (1) = .550, *p* = .458).

**Fig 3:**
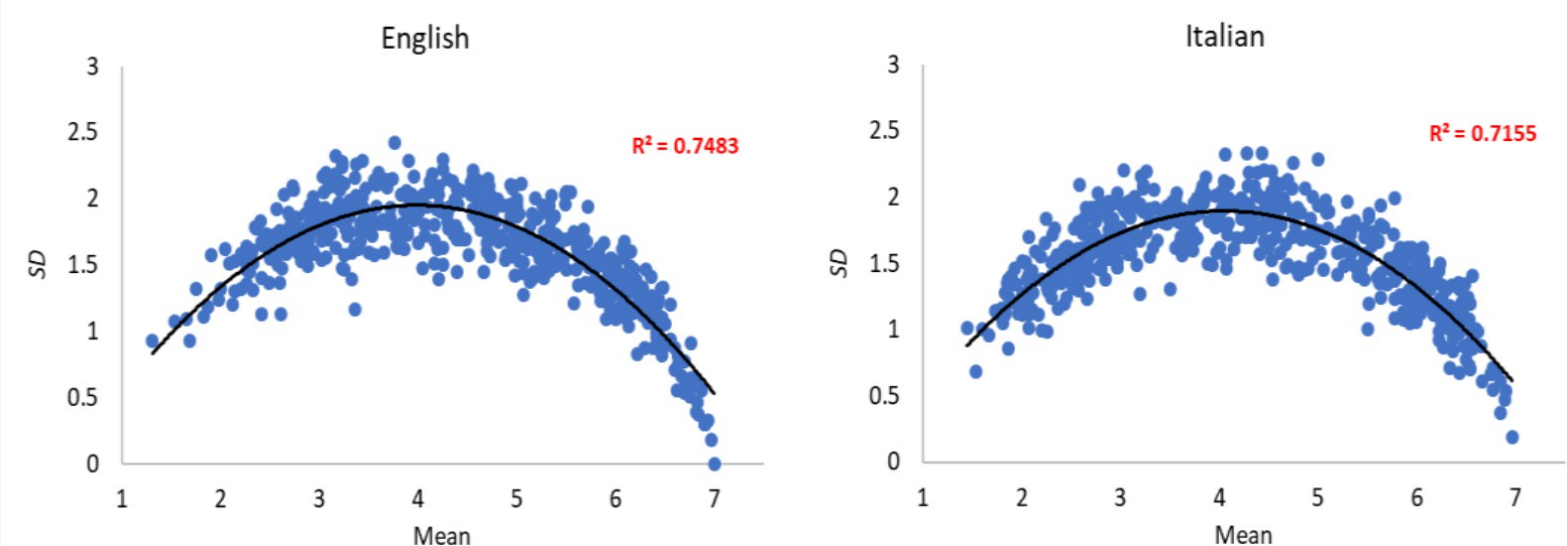
Homogeneity of the concreteness ratings. Standard deviations plotted against the respective means for English (left column) and Italian (right column) ratings.

Regarding the homogeneity of the participant ratings, Fig 3 shows the means of the ratings for each item sentence plotted against the corresponding standard deviations for both English and Italian. As can be seen, a global quadratic relation fits well with the data of both English and Italian participants. Therefore, item sentences that assume midrange values tended to receive more variable ratings across participants, suggesting that no sentences were consistently rated with midrange values of concreteness.

### Reliability of the measure

The consistency of the collected data was first evaluated by applying split-half correlations corrected with the Spearman-Brown formula after randomly dividing the participants into two subgroups of equal size. Reliability indexes were calculated on 2000 different randomizations of participants. The corrected split-half correlations were very high (English: median = .962, range = .954 –.970; Italian: median = .967, range = .962– .974), revealing that the resulting ratings were highly reliable and can be used in the Italian and English-speaking populations. An analysis of internal consistency was also performed using the Cronbach alpha coefficient. The ratings of each participant were used as variables and the individual sentences as cases following the procedure of [39, 40]. An alpha value was calculated for each stimulus list and participant sample (separately for English and Italian). The concreteness ratings of the participants showed high internal consistency within each language for each list, with Cronbach’s alpha coefficients ranging from.95 to.98 for English and from.94 to.98 for Italian, and the alpha values when each participant was dropped indicated that none of the participants would have increased reliability if they had been deleted (alpha coefficients from .94 to.98 for English and Italian languages).

### Relations among variables

Zero-order pairwise correlations showed that concreteness was related to the lexical density and accessibility of the sentence in both English and Italian. False Discovery Rate (FDR) correction was applied at *p* = 0.05, with the procedure described by Benjamini and Hochberg [64], to correct for multiple comparisons. In English, the word concreteness showed a small positive correlation with the lexical density (*r* = 0.098, *p* = 0.022) and a negative correlation with sentence accessibility (*r* = -0.122, *p* = 0.004). The same significant correlations were found for Italian (lexical density: *r* = 0.136, *p* = 0.001 and lexical sentence accessibility: *r* = -0.190, *p* < 0.001).

Furthermore, for the English language, we found small positive correlations with sentence length (*r* = 0.096, *p* = 0.026) and target lexical accessibility (*r* = 0.106, *p* = 0.014).

### Comparison with concreteness ratings of words in isolation

To assess our resource with respect to those in the literature, we compared the concreteness ratings collected for terms in isolation against the ratings collected in the CONcreTEXT norms. We did so for the English and Italian language, employing data provided by BRYS and MONT, respectively.

**Fig 4:**
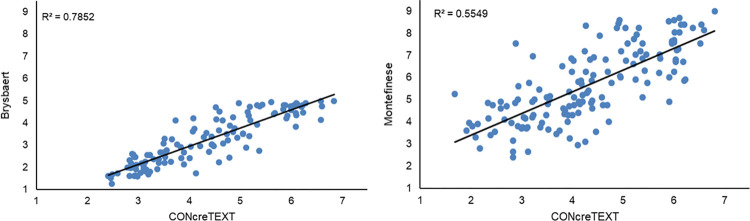
Comparison between concreteness ratings of words in sentences and in isolation. Mean ratings for concreteness in the present sample plotted against the corresponding mean ratings of the sample of Brysbaert, Warriner and Kuperman (2014; on the left), and Montefinese et al. (2014; on the right). Linear regression lines and the corresponding *R*^*2*^ values are shown for each plot.

Although we had to retrieve only one concreteness rating per term from the mentioned norms (where terms are rated once and in isolation), in our dataset each target term appears more than once (that is, a rating score was collected for each sentence containing the target term). We then compared our ratings with those in the literature by recording the correlation between the average of the values recorded in our norms and the value in either the BRYS or MONT data. In general, CONcreTEXT norms were significantly correlated to BRYS and MONT, respectively for English and Italian (see Fig 4). For English, we obtained a Spearman correlation (*ρ*) of 0.88 (*p* < 0.001); for Italian we obtained a *ρ* = 0.75 (*p* < 0.001).

### Comparison with WordNet synsets

We associated one or more meanings to each target term. In doing this, we relied on the WordNet [54] classification of meanings into sets of synonyms (i.e., synsets) that are used to refer to the same concept. Also based on the literature [65], we acknowledge in these regards that i) meanings might be at least partially overlapped rather than disjoint; and ii) the disambiguation process among meanings of the same word may result in a partial disambiguation, such that more than one meaning applies to the given context of occurrence of the target term; and iii) each target term admits more than one single meaning. The relationship between meanings and context is perhaps best understood if one considers word meaning as a probability distribution “over a set of latent senses and is modulated by context” [66]. For all mentioned reasons, we allowed for multiple semantic annotations, since target meanings are perhaps less rigidly separated than are WordNet synsets. Annotation of target words with word meanings was manually performed on the whole set of Italian and English sentences, to compare concreteness and polysemy, and to link concreteness scores to the meanings possibly conveyed for a given term through different sentences. To this aim, Open Multilingual WordNet (http://compling.hss.ntu.edu.sg/omw/) was used as the meaning inventory. The target word of each sentence was annotated with the best matching synset, according to the word meaning in the given context.

As expected, the annotators did not find a meaning for each occurrence since in some cases the set of available WordNet synsets does not cover the needed concept. Conversely, in other cases, a word was provided with multiple synset assignments, when annotators found more than one synset as suitable for the word in the given context. Incompleteness and multiplicity of synset annotations are common issues in this task [67]. In the current dataset, we observed a relevant percentage of both phenomena, and a strong remarkable difference between Italian and English. We found that a suitable meaning was not available for about 3% of English occurrences and for 8% of the Italian ones and multiple appropriate meanings for a word in context were found in 25% of Italian sentences and in 32% of the English dataset.

#### Agreement

To measure the inter-rater agreement, annotation has been performed in parallel by three authors on a subset of 20% of sentences in Italian and English. Sentences in which the target lemma had less than three synsets on Open Multilingual WordNet have been discarded: Although the number of synsets of a lemma should not have a significant impact on the agreement [67], a lemma with just one or two synsets could introduce a positive bias in the agreement value. Sentences with blank annotations, where at least one annotator did not find a suitable synset, have been filtered out from this group. Sentences with multiple annotations, where at least one annotator inserted more than one synset for the word in context, have been managed by creating two datasets with a unique synset per annotator in each sentence: in the *First* dataset, only the first annotated synset was reported; in the *Best* dataset, the combination of annotated synsets that maximize the agreement between annotators was selected. The agreement was calculated according to two measures, Fleiss *k* and Krippendorff *α*. The results are reported in Table 5.

**Table 5:** Study of the inter-rater agreement in the word meaning disambiguation task.

	First		Best	
	*k*	*α*	*k*	*α*
***Italian***	*** ***	*** ***	*** ***	*** ***
** Nouns**	0.464	0.465	0.49	0.491
** Verbs**	0.637	0.641	0.758	0.76
** Nouns + Verbs**	0.509	0.51	0.551	0.552
***English***	*** ***	*** ***	*** ***	*** ***
** Nouns**	0.764	0.765	0.776	0.777
** Verbs**	0.701	0.703	0.728	0.73
** Nouns + Verbs**	0.757	0.757	0.773	0.774

Human agreement in word meaning disambiguation tasks based on WordNet is highly dependent on the selected target words, and it is usually not high, with *k* values ranging from 0.3 to 0.8 [68–71]. Moreover, agreement differences between nouns and verbs are usually found, but they are not stable, and depend on the dataset in use.

The agreement measured on the current dataset is in line with the values reported in the literature for the same task on other datasets. A strong difference between Italian and English was observed, probably depending on a better-meaning distinction and coverage of the English WordNet. Agreement values in *Best* are higher than in *First*, with a standard deviation of 0.02 in English, and of 0.05 in Italian. The agreement differences between nouns and verbs are significant and completely unrelated between the two languages: the agreement value is higher for nouns in English, with a deviation of 0.06 (values computed in the *First* dataset), while in Italian a much higher agreement on verbs was obtained (*SD* = 0.17).

#### Comparison with concreteness ratings

**Fig 5:**
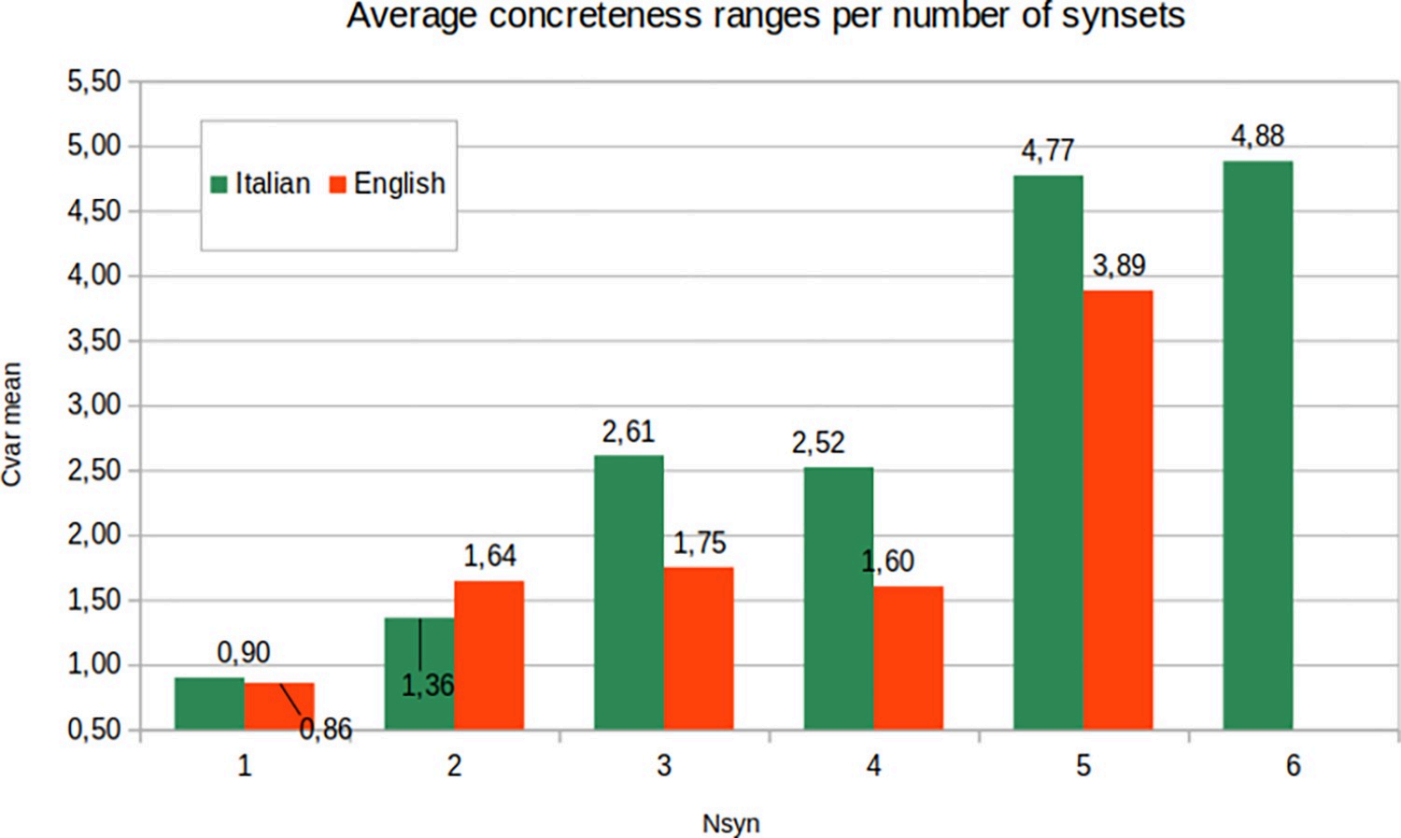
Comparison between synset annotation and concreteness ratings. Plot of the average concreteness ranges per number of synsets.

A comparison between synset annotation and concreteness ratings has been performed to see if and to what extent a variation of meanings in a target word is linked to a variation of the concreteness degree. To this aim, we counted the following numbers for each target lemma in our dataset, that is, the number of different synsets used in all the occurrences of a lemma (*Nsyn*); and the range of variation of the degree of concreteness, measured as the difference between the maximum and minimum concreteness rating (*Cvar*). Finally, we grouped the lemmas according to their *Nsyn* value and computed the mean *Cvar* of each group. In this way, we obtained a measure of variance for each group of lemmas with the same number of synsets in the data set. Results are reported in Fig 5.

The plot shows that, in fact, a higher number of meanings underlying a given lemma are associated with a higher deviation in the concreteness ratings. For example, lemmas where all the occurrences belong to the same synset have, on average, a variation of concreteness values below 1.0, both in Italian and in English; lemmas with 3 represented synsets have an average *Cvar* of 2.61 for Italian and 1.75 for English; lemmas with 5 different synsets report a stronger variation of concreteness levels: 4.77 in Italian and 3.89 in English. Interestingly, in English we do not have any lemma with 6 different synsets.

The correlation between *Nsyn* and *Cvar* is measured with Kendall’s τ and reports a significant positive correlation: τ = 0.41 for the Italian dataset and τ = 0.34 for the English dataset (*p*s < .01). These results show that when a lemma is used with only one meaning, it tends to have a more stable concreteness degree; conversely, a wider variability in concreteness ratings is observed when a lemma is used with different meanings.

We deepened our analysis on the standard deviation associated with the average concreteness scores of words with one sense vs words with multiple senses. Even though we observe an increasing difference between highest and lowest concreteness score as the number of senses grows, the standard deviation associated with terms with one sense is only slightly lower than that associated with polysemous terms (1.62, 1.63 and 1.48, 1.53 for the English and Italian language, respectively). Our results show that the variability in the identification of the underlying sense is similar for targets, irrespective of whether these had one or more annotated senses. Such a hypothesis seems to be confirmed by the ratio between standard deviation and concreteness average scores: this figure is .37 and .43 for terms occurring with one sense and polysemous terms (English), and .42 and .43 for Italian terms, respectively. No information was provided to our raters on whether a word was polysemous or not.

### Differences between nouns and verbs in concreteness ratings

We investigated differences between the concreteness ratings provided for target nouns and verbs in each sentence by both Italian and English participants. To this end, we performed a series of two-tailed independent *t*-tests. We found that the average concreteness ratings of Italian speakers were not significantly different between verbs and nouns (*M* = 4.21 and 4.30, *SD* = 1.40 and 1.58, respectively; *t*(560) = .677; *p* = .498), and the effect size was very small (Hedges’ *g* = .059). The same result emerged for English ratings, with no differences between nouns and verbs (*M* = 4.50 and 4.37, *SD* = 1.48 and 1.24, respectively; *t*(542) = 1.041; *p* = .298; *g* = .093). Furthermore, in order to investigate whether there was a difference in the rating variability between nouns and verbs for each language, we performed the *t* tests on their standard deviations. While the results did not show any difference for the Italian sentences (*M* = 1.52 and 1.55, *SD* = 0.37 and 0.33, for nouns and verbs, respectively; *t*(560) = .952; *p* = .342; *g* = .084), a significant difference has been found for the English sentences (*t*(542) = 2.203; *p* = .028), with a higher variability for verbs (*M* = 1.66, *SD* = 0.43) compared to nouns (*M* = 1.59, *SD* = 0.32), although the effect size was small (*g* = .178).

## Discussion

The present study has proposed a set of newly collected contextual concreteness ratings that substantially extends with further ratings and lexical measures the data released for the shared task CONcreTEXT@Evalita [33]. These norms were obtained through an online procedure whereby English and Italian speakers were asked to assign a concreteness score to over a hundred words, each occurring in different sentence contexts. Indeed, although we cannot compare the concreteness ratings of the two languages directly, results from each language show a relatively similar pattern (in terms of data reliability, and correlational patterns).

The key asset of these norms is that the sentences have been obtained from the WikiHow website as natural, ecological, examples of real-world language. This aspect makes the current concreteness norms easily extensible to other languages.

An exploration of the distribution of concreteness ratings revealed that their distribution deviated significantly from normality. Furthermore, by plotting the mean and standard deviation of the ratings, the results showed a quadratic function, suggesting that item sentences that assume midrange values tended to receive more variable ratings across participants.

We tested the reliability of our ratings, which was checked in depth and through different measures, obtaining high figures, well above 0.9, both in the split-half tests and in the Cronbach’s alpha coefficients in both English and Italian languages. This pattern of results indicates that our ratings have high internal consistency, and thus they provide an optimal estimate of how participants perceive the meanings delivered by target terms in context based on their perceptual grounding and language.

Importantly, different from existing work [1, 2, 48] our ratings were collected in the more ecological setting of words in context. In addition, concreteness scores were collected for a set of differing contexts. This approach allows for a more precise annotation of a term based on its specific context, for example, allowing one to distinguish among the different meanings of the word ’field’ based on its context, such as ’wheat fields’, ’magnetic fields’ and ’fields of expertise’, where each occurrence of ‘field’ is equipped with its own specific concreteness content. Contextual annotation thus involves a hidden task, that is, the identification of the meanings at the base of the concreteness rating (more on the meaning-identification task can be found in [49]). All targets were then meaning-annotated according to WordNet and are part of the released data. Although a high correlation was observed between concreteness ratings in context and in isolation, we observed a significant role of the number and types of meanings in the ratings in context. This is a clear effect of the contextual nature of meanings and can be interpreted as the main proof in favor of this work, where concreteness is seen as a function of (a specific meaning in) a given context. Further studies should consider the role of meaning dominance (i.e., the existence of a predominant meaning for a given term) on ratings of words in isolation and its role in explaining the variation between ratings in isolation and in context.

Word concreteness is an intriguing research topic area at the intersection of psychology, linguistics, lexical semantics, computational modeling, and NLP. In the last few years, it has attracted growing research efforts since it impacts how we acquire, access, and make use of word meanings. For the NLP community, the present norms may be relevant for building meaning embeddings that are indexed on both terms and meanings, such as DeConf [72], LSTMEmbed [73], LessLex [74]; and, to build contextual embeddings, such as LMMS [75] and SensEmBERT [76]. More generally, the present norms can be employed to acquire and refine language models by adding information on word concreteness, thereby resulting in richer and more precise representations.

Although the number of annotated occurrences is limited and not all term meanings are covered, the present work offers a first investigation reporting on the role of context in concreteness ratings in two languages (English and Italian) and introduces a methodology for data collection that has proven to be solid. Following this methodology, the data set can be easily expanded in the future, about the number of target words, contextual sentences per target word, and additional languages. This will contribute to promote the generalizability of studies on the concreteness effect (and to drive a more overarching theory of semantic representation) since our understanding of this effect has been disproportionally informed by English native speakers. Future research should also take into account the homonymous words, which are currently underrepresented within our dataset. Indeed, the different meanings of homonymous words can vary in concreteness, and the context is essential to select the exact meaning of homonymous words as well as of polysemous words. Therefore, increasing the inclusion of homonymous words in future normative studies will allow investigations on the intricate interplay between concreteness, context and word ambiguity, shedding new light on the dynamics of language processing.

## Conclusion

In summary, we collected word concreteness norms in more than 1000 English and Italian sentences. The high consistency of the collected data has been demonstrated by the high split-half correlations and the Cronbach alpha coefficients. We also showed a significant correlation between the concreteness value of a target word and the number of different meanings in which it is encountered, confirming a link between word concreteness and context. We believe that CONcreTEXT norms are a valuable source of information and can be used confidently for the selection of words in different contexts in future research, despite its small size. We hope indeed, that these norms will be useful to the research community. As far as we know, ours are the first concreteness norms for words in context sentences for English and Italian. As such, these norms should be useful to researchers working with these languages or interested in cross-linguistic comparisons.
